# Drink driving among Aboriginal and Torres Strait Islander Australians: What has been done and where to next?

**DOI:** 10.1111/dar.13418

**Published:** 2021-12-19

**Authors:** Michelle S. Fitts, Richard Burchill, Scott Wilson, Gavan R. Palk, Alan R. Clough, Katherine M. Conigrave, Tim Slade, Anthony Shakeshaft, K. S. Kylie Lee

**Affiliations:** ^1^ Institute for Culture and Society Western Sydney University Sydney Australia; ^2^ Menzies School of Health Research, Charles Darwin University Alice Springs Australia; ^3^ James Cook University Australian Institute of Tropical Health and Medicine Cairns Australia; ^4^ Jabalbina Yalanji Aboriginal Corporation Mossman Australia; ^5^ Aboriginal Drug and Alcohol Council SA Aboriginal Corporation Adelaide Australia; ^6^ Faculty of Medicine and Health Discipline of Addiction Medicine, NHMRC Centre of Research Excellence in Indigenous Health and Alcohol, The University of Sydney Sydney Australia; ^7^ The Edith Collins Centre (Translational Research in Alcohol Drugs and Toxicology) Sydney Local Health District Sydney Australia; ^8^ Australian Institute for Suicide Research and Prevention, School of Applied Psychology Griffith University Brisbane Australia; ^9^ Drug Health Services Royal Prince Alfred Hospital Sydney Australia; ^10^ Faculty of Medicine and Health The Matilda Centre for Research in Mental Health and Substance Use, The University of Sydney Sydney Australia; ^11^ National Drug and Alcohol Research Centre UNSW Sydney Sydney Australia; ^12^ Faculty of Health Sciences National Drug Research Institute, Curtin University Perth Australia; ^13^ Centre for Alcohol Policy Research La Trobe University Melbourne Australia; ^14^ Burnet Institute Melbourne Australia

**Keywords:** alcohol, drink driving, Aboriginal and Torres Strait Islander people, Indigenous, remedial program

## Abstract

The Australian Government will set the direction for addressing road safety over the next decade with its 2021–2030 National Road Safety Strategy. This road map will detail objectives and goals agreed upon by all Australian states and territories. Similar to previous national strategies, Aboriginal and Torres Strait Islander (Indigenous) Australians are a high priority population. Indigenous Australians are over‐represented in serious injury and fatal road crashes, with alcohol a leading factor. Therapeutic and educational programs are a major strategy among the suite of measures designed to reduce and prevent drink driving in Australia. The release of this new strategy provides a timely opportunity to reflect on what is known about drink driving among Indigenous Australians and to consider the suitability of existing therapeutic and educational drink driving programs for Indigenous Australian contexts. Here, we summarise factors that contribute to drink driving in this population and identify outstanding knowledge gaps. Then, we present an overview of drink driving programs available for Indigenous Australians along with suggestions for why tailored programs are needed to suit local contexts. The response to address drink driving among Indigenous Australians has been fragmented Australia‐wide. A coordinated national response, with ongoing monitoring and evaluation, would improve policy effectiveness and inform more efficient allocation of resources. Together this information can help create suitable and effective drink driving programs for Indigenous drivers and communities Australia‐wide.

## Introduction

Indigenous peoples in Australia, Aotearoa/New Zealand and the USA experience higher levels of road trauma than their non‐Indigenous counterparts [1, 2, 3, 4, 5, 6]. In Australia, road crashes are responsible for more than a quarter (27.1%) of the total injuries annually sustained by Aboriginal and Torres Strait Islander (Indigenous) peoples [7]. Compared with other Australians, fatal road crashes are three times higher among Indigenous Australians (20.1 vs. 7.4 deaths per 100 000) [8]. A key factor in this trauma is alcohol [1, 2, 3]. ‘Drink driving’ in Australia is defined as driving above the legal alcohol threshold of 0.05 g/100 ml for a driver with a full licence [9]. In the Australian state of Queensland, illegal levels of alcohol were present four times as often in road crashes involving Indigenous drivers compared with other drivers (24.5% vs. 5.6% respectively; 2001/2002–2005/2006) [2]. A higher rate of Indigenous drink driving arrests is also reflective of this, compared with non‐Indigenous counterparts (3776 vs. 582 per 100 000) in Western Australia [3]. Alcohol‐related road trauma has been a longstanding concern for Indigenous communities Australia‐wide [10, 11]. Community leaders have led action to address drink driving and road safety [12, 13].

In Australia, the National Road Safety Strategy (NRSS) lays out agreed objectives and goals for all Australian states and territories [9, 14, 15, 16, 17]. The development of drink driving programs for and with Indigenous Australians was one aim of the last strategy [14]. In 2021, the Australian Government's Office of Road Safety will release the NRSS for the next decade (2021–2030) [18]. Again, Indigenous Australians are a priority group [18, 19, 20]. It is, therefore, timely to reflect on what is known about Indigenous Australian drivers who drive while above the legal alcohol threshold; what drink driving programs have been developed during the previous NRSS for this population [14]; and why some existing Australian drink driving programs may not be typically suitable for Indigenous Australian contexts. We then suggest three key considerations likely to support the development and implementation of more effective drink driving programs for Indigenous peoples Australia‐wide.

## What Do We Know about Indigenous Australians Who Drink and Drive?

Indigenous people are more likely to drink and drive if they are in non‐urban areas [21], are aged less than 25 years [21] and have multiple drink driving convictions [22, 23]. Specifically, Indigenous drink drivers from remote areas [24] are more likely to re‐offend and to record a higher range blood alcohol concentration (≥0.15 g/100 ml in Australia) [25]. Many contributing factors for drink driving are unique to the Indigenous Australian context [26, 27, 28, 29]. For example, cultural obligation to drive (even after drinking) has been shown to be a major factor, especially in regional and remote contexts [26, 27, 28, 29]. Young people, in particular, can experience pressure from family or older, respected community members to drive after drinking.The tightening of local alcohol restrictions [30] has had a mixed impact on drink driving [26]. In some remote commnities, it has led to increases in drink driving through consumption of ‘sly grog’ outside of the community [26]. Also, Indigenous Australians who drink and drive describe feeling that they are able to manage the road and apprehension‐related risks [27]. For example, some drinkers will nominate an individual to drive based on who is least intoxicated or who has the least number of prior convictions [27]. Finally, movement between and within rural and remote areas is required for Indigenous peoples to maintain cultural obligations, community connections and access services, which can often require travel of vast distances (e.g. hundreds of kilometres). Yet, few alternative transport options are available in these settings and those that are can be expensive or limited (e.g. access to taxis or public transport that is only offered at particular times of the day or on certain weekdays) [12].

Despite these observations, existing information on Indigenous Australians who drive above the legal alcohol limit is fragmented, with qualitative data only available from a small number of towns and communities within three of eight Australian jurisdictions (New South Wales, Queensland and Western Australia) [3, 22, 23, 25, 26, 27, 28, 29, 30, 31, 32]. Drink driving is the responsibility of and enforced by individual state and territory governments. Drink driving data are therefore managed by individual states and territories, and the extent these data are reported on varies. For example, some studies and government reports present overall Indigenous drink driving rates only by jurisdiction [3] or by recidivism rates and associated demographic and offence characteristics [22, 23, 25]. One study compared Indigenous and non‐Indigenous drink drivers [31], while another study compared Indigenous first‐time convicted and Indigenous repeat drink drivers [25]. This makes it difficult to determine regions of high risk and impact of policy and programs on drink driving. In order to have effective drink driving programs for Indigenous Australians, more research is needed about the socio‐cultural and environmental characteristics of Indigenous peoples who drink and drive across all Australian jurisdictions, the patterns of drinking among individuals who drink and drive and the differences across urban, regional and remote regions [12].

## Why Do Australian Drink Driving Programs Need Re‐Focusing?

Under the previous NRSS (2011–2020) [14], a pilot community program was designed with four Far North Queensland communities and one northern New South Wales regional town as well as resources developed for Indigenous Australians who drink and drive in Western Australia. The community program and resources to support drink driving education were developed in partnership with researchers, state governments and communities [26, 32, 33]. The community program was designed to be delivered on country with individuals who drink and drive, community members and community leaders [32]. The program treated drink driving as a social and community phenomenon, with focus placed on positive alternatives to drinking and driving (e.g. cultural healing of trauma that may lead to drinking and re‐arranging individual lifestyles to promote healthy living) [12, 32]. Despite these promising advancements, a recent review of Australian drink driving programs identified most programs operating at present are for the general population [9].

Current drink driving programs developed for general populations [9, 34, 35, 36, 37] can be unsuitable for many Indigenous Australians for several reasons. Firstly, program content and delivery are focused on the individual convicted of the offence only (as opposed to taking a holistic view of the individual in the context of their family and community) [38, 39]. In keeping with this, program content has been developed with the aim of modifying the conscious decision making and planning of individuals convicted of drink driving [40]. Absent from these programs is a consideration of the Indigenous contexts in which drinking and driving occurs (e.g. cultural obligations, presence of alcohol restrictions), and the need to accommodate attitudes that affect decision making at family and whole community levels [26, 27, 28].

Secondly, unlike drink driving programs in other similarly colonised countries [41, 42, 43, 44, 45], many drink driving programs have not drawn from the strengths of Indigenous cultural values, practices and family (e.g. a bicultural approach to care). In contrast, drink driving programs from the USA and Aotearoa/New Zealand encourage Indigenous participants to take part in talking circles and sweat lodge ceremonies [43, 45], to devise ways of observing cultural protocols without drink driving [41] and to promote program participation by family members [42]. These programs have been shown to reduce recidivism [43] and frequency of drinking [45].

Thirdly, tools used by Australian drink driving programs, such as Alcohol Use Disorders Identification Test‐Consumption [46], help screen for risky drinking. Such tools are not designed to collect a detailed history about drinking patterns that may be useful to inform drink driving programs in Indigenous contexts [47]. For example, Alcohol Use Disorders Identification Test‐Consumption's frequency response categories assume drinking regularity [47]. Yet, episodic drinking with extended ‘dry’ periods is common for some Indigenous Australians who do drink alcohol [48]. Sharing of drinks and drinking in non‐standard containers [49], limited awareness of what constitutes a ‘standard drink’ (10 g each of ethanol) and living in regions with intermittent alcohol access also present challenges in how self‐reported patterns of drinking are accurately obtained [50]. Tools that enable participants to reflect on drinking using tools validated for Indigenous Australian contexts [51, 52, 53], combined with Indigenous‐specific cut‐off scores [54], would better enhance our understanding of their patterns of drinking, and drinking context, to inform program content.

## Suggestions to Inform Drink Driving Programs Designed with and by Indigenous Australians

Just a few original drink driving programs and resources have been developed for Indigenous Australians, despite this group being a priority population at a national level [14, 15, 16, 17, 18]. The delays stem from a combination of lack of coordination in drink driving data collection and several research gaps. While communities and individual states and territories are likely to have differing drink driving priorities, three steps could help improve the evidence used to inform drink driving programs for Indigenous Australians.

1. *A national approach to collect and analyse conviction data* is needed – that involves ongoing monitoring to identify high‐risk regions and to increase our understanding of individuals' and communities' characteristics associated with drink driving. At present, available data on Indigenous Australians who are convicted of drink driving are both outdated and not reported nation‐wide. In keeping with this, systematic assessment of local alcohol restrictions and their impact on drink driving is also needed [55, 56]. Currently, most evaluations only present anecdotal evidence on such impacts [57, 58, 59, 60].
2. *Improved understanding is needed of the context of driving after drinking among Indigenous Australians* through more research on socio‐cultural and environmental characteristics. As identified here, just a small number of Indigenous communities have been involved in studies on the context of drink driving [26, 27, 28, 29]. Data on patterns of drinking among Indigenous Australians who drink and drive could also help inform drink driving program content. Improved approaches and tools validated for Indigenous Australian contexts have been developed [51, 52] that enhance self‐report of alcohol use behaviours among Indigenous Australians. Reliance on blood alcohol readings is useful but does not offer insight into patterns of drinking (e.g. median standard drinks per drinking occasion – that takes into account episodic drinking, sharing of drinks, drinking from non‐standard containers) [51, 60].
3. *Co‐design partnerships* between local communities with government, non‐government agencies and researchers to help create target action areas, distinctive messages and strategies for each community and region. These partnerships would also provide foundation upon which lessons can be shared across regions. There is a growing evidence base supporting community‐led programs as the most successful approach for health improvement. The opportunity to create more programs to reduce drink driving harm could leverage the evidence from existing systematic reviews of general alcohol harm reduction programs experienced by Indigenous Australians [61] and replicate community‐based partnership approaches to reducing alcohol harm [62]. Evaluation of new strategies, policies and programs are vital to determine what impact these measures have on drink driving.

## Conclusion

Despite being a priority group under the last NRSS (2011–2020) [14], progress to design and implement programs for Indigenous Australians who drink and drive has been piecemeal [32, 33]. Drink driving programs could be strengthened by incorporating cultural concepts and family participation [41, 42, 43, 44, 45]. We identified target areas that would be needed to directly address the gaps in this under‐researched area of alcohol harm. Greater research investment, together with meaningful partnerships between community, researchers and government will be key to achieving this.

## Conflict of Interest

The authors have no conflicts of interest.
